# Safe administration of ipilimumab plus nivolumab to a dialysis patient with renal cell carcinoma

**DOI:** 10.1002/iju5.12231

**Published:** 2020-10-28

**Authors:** Takuya Iwaki, Aya Niimi, Masato Kano, Yoshiaki Kurokawa, Uran Yoshizaki, Keina Nozaki, Akira Nomiya, Hideyo Miyazaki, Haruki Kume

**Affiliations:** ^1^ Department of Urology Center Hospital of the National Center for Global Health and Medicine Toyama Shinjuku‐ku Tokyo Japan; ^2^ Department of Urology New Tokyo Hospital Chiba Japan; ^3^ Department of Urology The University of Tokyo Hospital Tokyo Japan

**Keywords:** dialysis, end‐stage renal disease, immune checkpoint inhibitor, ipilimumab, nivolumab, renal cell carcinoma

## Abstract

**Introduction:**

The combination of ipilimumab plus nivolumab has been used as first‐line therapy for metastatic renal cell carcinoma. While it is well known that hemodialysis patients have a higher rate of renal cell carcinoma compared to the general population, no reports have described the safety of ipilimumab–nivolumab in metastatic renal cell carcinoma patients on hemodialysis.

**Case presentation:**

A 73‐year‐old man with a 21‐year history of dialysis was referred to our department in 2019 for bilateral renal tumors and multiple lung nodules. He had already been diagnosed with bilateral renal tumors in 2015, without undergoing surgery due to comorbidities. In May 2019, contrast‐enhanced computed tomography revealed multiple lung metastases in addition to the existing renal tumors; consequently, he was treated with four doses of nivolumab–ipilimumab with no adverse events.

**Conclusion:**

The combination of ipilimumab plus nivolumab was safely used in a hemodialysis patient with metastatic renal cell carcinoma.

**Abbreviations & Acronyms**
CT: computed tomography
ESRD: end‐stage renal disease
ICI: immune checkpoint inhibitor
irAE: immune‐related adverse event
mRCC: metastatic renal cell carcinoma
RCC: renal cell carcinoma

**Keynote message**

The combination of ipilimumab plus nivolumab has been used as first‐line therapy for intermediate‐ or poor‐risk advanced RCC. While RCC is an important complication in dialysis‐dependent patients, there is lack of evidence describing the tolerability or efficacy of ipilimumab or nivolumab in these patients. This could potentially lead to medication denial or undertreatment in mRCC patients receiving dialysis. This is the first reported case of combination therapy with ipilimumab plus nivolumab for mRCC in patients on dialysis.

## Introduction

The immunotherapy combination of ipilimumab (an anti‐CTLA‐4 antibody) plus nivolumab (a PD‐1 ICI antibody) has become widely used as first‐line treatment for intermediate‐ or poor‐risk patients with advanced RCC ever since the results of the phase 3 CheckMate 214 trial were released.^1^

Dialysis patients are known to have a higher incidence rate of RCC than do the general population;^2^, ^3^, ^4^ nevertheless, there have so far been no controlled clinical studies to examine the efficiency and safety of ipilimumab plus nivolumab in hemodialysis patients with mRCC. Although the U.S. Food and Drug Administration prescribing information for ipilimumab and nivolumab describes that no dose adjustment is needed for patients with renal impairment, there exists no specific information regarding patients on dialysis.^5^, ^6^ This uncertainty over drug dosing/pharmacokinetics and the lack of efficacy and safety data could potentially lead to medication denial or undertreatment in mRCC patients receiving dialysis. While there are several case reports suggesting the efficacy and safety of monotherapy with either ipilimumab or nivolumab in dialysis patients^7^, ^8^, ^9^, ^10^, ^11^, ^12^ to the best of our knowledge, no case of combination therapy with ipilimumab and nivolumab has been reported yet.

## Case presentation

**Fig. 1:**
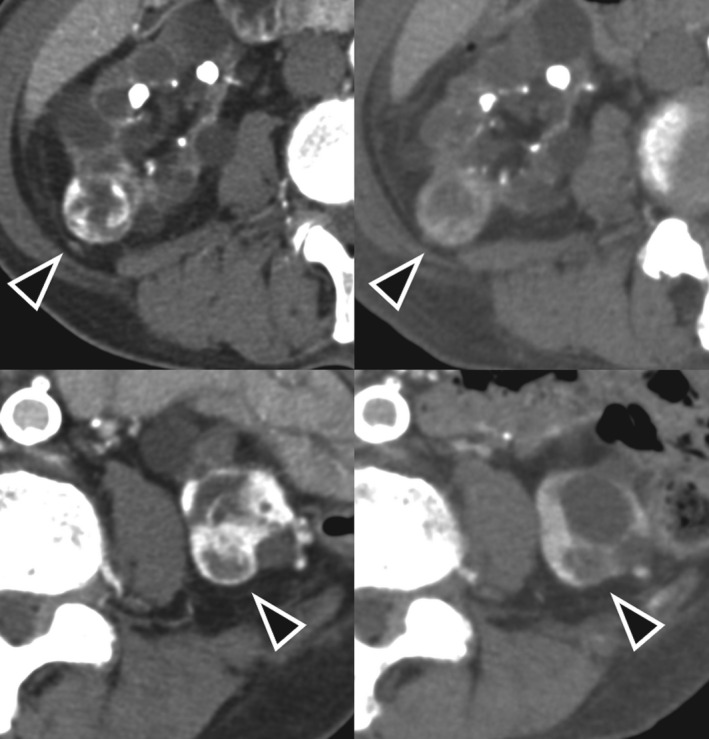
Contrast‐enhanced CT of the renal tumors before combination therapy with ipilimumab plus nivolumab (upper left: right kidney tumor; lower left: left kidney tumor) and after 6 months of treatment (upper right: right kidney tumor; lower left: left kidney tumor). No remarkable changes are observed

**Fig. 2:**
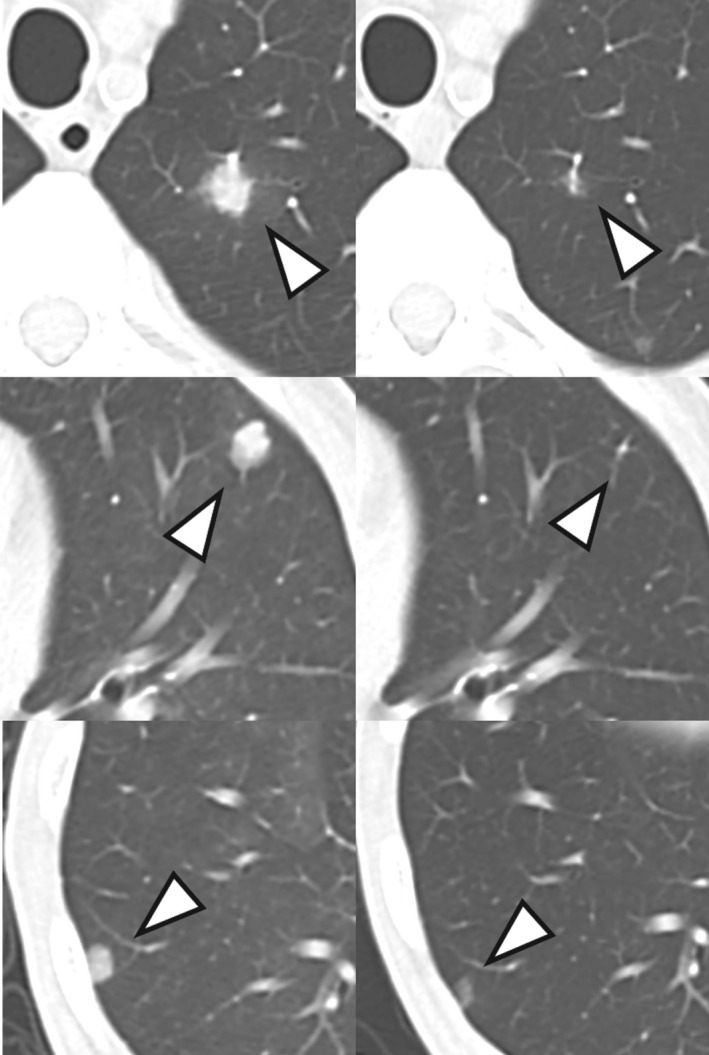
Chest CT before combination therapy with ipilimumab plus nivolumab (left) and after 6 months of treatment (right). The lung metastases have shrunk

A 73‐year‐old man with bilateral RCC and multiple lung metastases was referred to our department in May 2019. He had been receiving dialysis since 1999 due to end‐stage kidney dysfunction induced by diabetes. He had also been diagnosed with bilateral renal tumors at his previous hospital in 2015, when he was first scheduled for bilateral nephrectomy; later on, however, the operation was cancelled owing to his comorbidities (i.e. hypotension and cardiac arrhythmia), and therefore, he was kept under observation for three and a half years until he developed an acute myocardial infarction in May 2019, at which time a CT scan revealed multiple lung nodules. Moreover, during the period of careful observation, he had experienced multiple strokes, without undergoing any biopsy procedures because of his inability to stop anticoagulation therapy. We clinically diagnosed the patient with multiple lung metastases of bilateral RCC, since no other malignancies were noted on radiological examination. We subsequently started treatment with ipilimumab plus nivolumab in January 2020. Both of his renal tumors were classified at T1aN0M1 and were classified as intermediate risk according to the International Metastatic RCC Database Consortium risk classification since it met one factor, which was anemia. He received ipilimumab (1 mg/kg) plus nivolumab (240 mg/body) every 3 weeks for four cycles with no major adverse events, followed by nivolumab monotherapy (240 mg/body) every 2 weeks. Six months after treatment, contrast‐enhanced CT showed no change in the size of the bilateral renal tumors but a reduction in the lung metastasis (Figs 1 and 2). He still continues to be treated with nivolumab and has suffered no major adverse events, including irAEs.

## Discussion

In the phase 3 CheckMate 214 trial, ipilimumab plus nivolumab showed superior efficacy over sunitinib in patients with intermediate‐ or poor‐risk advanced RCC with a clear‐cell component.^1^ In Japan, ipilimumab plus nivolumab was approved by Japanese National Health Insurance as first‐line treatment for mRCC in August 2018.

Since the first report by Dunnill *et al.* in 1977, RCC has been reported to occur at a higher incidence rate in dialysis patients than in the general population.^2^, ^3^, ^4^, ^13^ While RCC is an important complication in dialysis‐dependent patients, no controlled clinical trials have been conducted to evaluate the efficacy and safety of ipilimumab or nivolumab in these patients. The CheckMate 214 trial excluded patients with impaired renal function and therefore failed to take notice of the efficacy and safety of ipilimumab combined with nivolumab in patients on dialysis. Additionally, a thorough search of ClinicalTrials.gov revealed no planned, ongoing, or completed trials investigating the safety and efficacy of ipilimumab or nivolumab in patients with renal impairment or those undergoing dialysis. There are some case reports suggesting the safety and efficiency of monotherapy with either ipilimumab or nivolumab in dialysis patients.^7^, ^8^, ^9^, ^10^, ^11^, ^12^ In a study by Cheun *et al.*, for instance, 10 of 13 dialysis patients responded to ICIs.^10^ However, to the best of our knowledge, there are no case reports of nivolumab plus ipilimumab combined immunotherapy in dialysis patients with mRCC. Hence, this is the first reported case of combination therapy with ipilimumab plus nivolumab for mRCC in patients on dialysis.

ESRD patients have impaired immunity, due to reduction of activities of immune system cells (B cell, T cell, monocytes, macrophages) and lower antibody levels.^14^, ^15^, ^16^ Ipilimumab and nivolumab are monoclonal antibodies that inhibit the expression of proteins and thereby enhance activities of T cell against cancer. Theoretically, these drugs might have a low efficacy in the treatment of cancer in dialysis patients. However, in a study by Cheun *et al.*, 10 of 13 dialysis patients responded to ICIs.^10^ Therefore, ICIs can be beneficial to treat cancer in ESRD patients undergoing dialysis, while there might have been publication bias.

In population pharmacokinetic studies, renal impairment has been proven to have no clinically important effect on the clearance of ipilimumab and nivolumab, which are unlikely to be removed by dialysis because of their high molecular weights and are expected to be cleared by proteolytic degradation through receptor‐mediated endocytosis or nonspecific endocytosis mainly in hepatic or reticuloendothelial cells.^5^, ^6^, ^13^ The USA (U.S. Food and Drug Administration) and Japan (Pharmaceuticals and Medical Devices Agency) prescribing information for ipilimumab and nivolumab describe that no dose adjustment is required for patients with renal impairment; yet, no specific information is available on patients receiving dialysis.^5^, ^6^ Because of the lack of efficacy or tolerability data, oncologists might be reluctant to use ICIs in dialysis patients. Previous case reports have recommended no dose adjustments of ipilimumab and nivolumab for mRCC in dialysis‐dependent patients.^7^, ^8^, ^9^, ^10^, ^11^, ^12^ In our case, the patient was able to complete four cycles of ipilimumab plus nivolumab, with neither dose adjustment nor major adverse events (e.g. irAE).

Major oncologic societies have published clinical guidelines of irAE management.^17^, ^18^, ^19^ However, no guidelines discuss about the management of irAE for dialysis patients. In the report of Cheun *et al.*, one ESRD patient developed symptomatic grade 2 immune‐related pneumonitis, which improved with the cessation of nivolumab and steroid treatment for 1 month.^10^

This case report has two limitations. First, no biopsy was performed in this patient because of his inability to stop anticoagulation therapy. Second, the sample size was too small to make conclusions regarding the safety and efficacy of ipilimumab plus nivolumab in hemodialysis patients with mRCC and larger studies are needed.

We herein report a case of combination therapy with ipilimumab and nivolumab for mRCC in a hemodialysis patient with ESRD without any adverse events.

## Conflict of interest

The authors declare no conflict of interest.
